# Modulation of translation and induction of autophagy by bacterial exoproducts

**DOI:** 10.1007/s00430-012-0271-0

**Published:** 2012-09-19

**Authors:** Gisela von Hoven, Nicole Kloft, Claudia Neukirch, Sabrina Ebinger, Wiesia Bobkiewicz, Silvia Weis, Klaus Boller, Kim D. Janda, Matthias Husmann

**Affiliations:** 1 Institute of Medical Microbiology and Hygiene, University Medical Center, Johannes Gutenberg-University Mainz, Hochhaus am Augustusplatz, 55131 Mainz, Germany; 2 Department of Immunology, Paul Ehrlich-Institute, Morphology Section, 63225 Langen, Germany; 3 Department of Chemistry and the Skaggs Institute for Chemical Biology, The Scripps Research Institute, 10550 North Torrey Pines Road, La Jolla, CA 92037 USA

**Keywords:** Membrane damage, Translation, Autophagy, Quorum-sensing hormone, Pore-forming toxins

## Abstract

Autophagy is a catabolic process of paramount importance for cellular homeostasis during starvation. Generally, autophagy and translation are inversely regulated. Many kinds of stress lead to attenuation of translation *via* phosphorylation of eukaryotic translation initiation factor alpha (eIF2α). This response is conserved from yeast to man and can be either protective or detrimental depending on strength and duration of stress, and additional factors. During starvation or viral infection, phosphorylation of eIF2α is required for induction of autophagy. As exemplified here by α-hemolysin, a small pore-forming toxin (PFT) of *Staphylococcus aureus* and (S)-3-oxo-C12-homoserine lactone [(S)-3-oxo-C12-HSL], a quorum-sensing hormone of *Pseudomonas aeruginosa*, bacterial exoproducts may also impact translation and autophagy. Thereby, PFT and (S)-3-oxo-C12-HSL act differentially. Damage of the plasma membrane by PFT causes efflux of potassium, which leads to amino acid starvation and energy loss. This triggers amino acid-sensitive eIF2α-kinase GCN2, as well as energy sensor AMPK, and deactivates mTORC1. The output of this response, that is, transient metabolic reprogramming is an essential part of a defense program which enables cells to survive attack by a pore-forming agent. Thus, nutrient/energy sensors serve as sentinels of plasma membrane integrity. In contrast to PFT, (S)-3-oxo-C12-HSL does not cause acute loss of ATP or activation of GCN2, but also triggers phosphorylation of eIF2α and inhibits translation. This response appears not to depend on efflux of potassium and requires eIF2α-kinase PKR. Like α-toxin, (S)-3-oxo-C12-HSL increases lipidation of LC3 and accumulation of autophagosomes in cells. Apart from directly affecting host-cell viability, bacterial exoproducts might galvanize bystander cells to prepare for close combat with microbial offenders or inadvertently accommodate some of them.

## Basic mechanisms of autophagosome formation

The term autophagy—hereafter used in place of “macroautophagy”—was first introduced during the CIBA foundation symposium on lysosomes, London, in 1963 [1]. It denotes an important catabolic process in eukaryotes, the hallmark of which is the formation of an intracellular double-bilayer membrane compartment, the autophagosome. Autophagy may occur in an apparently random manner anywhere in the cytosol, or alternatively at particulate cargo destined to be degraded or otherwise removed from a cell. The principal function of autophagy is break down and recycling of macromolecules, but there is evidence for involvement in atypical secretion as well [2–5]. Autophagic flux is subject to complex regulation by extracellular and intracellular cues to meet cellular energy requirements, provide molecular building blocks, and achieve “garbage” removal.

Although the source of the membrane required for the formation of an autophagosome remains a matter of debate, there is evidence that it may be derived from various organelles, including ER, mitochondria, nucleus, Golgi, and PM [6]. The formation of closed, vesicular autophagosomes is preceded by the occurrence of crescent-shaped double-membrane phagophores. Autophagosome biogenesis is commonly subdivided into three steps: initiation or nucleation, elongation, and maturation. Genetic studies in yeast have led to the identification of more than 30 genes involved in this process, and mammalian homologs are known for many of them [7, 8].

The class III phosphoinositide 3 kinase (PI3K) Vps34, which acts in complex with additional proteins including Beclin1 and Atg14L/Barkor catalyzes the formation of PI(3)P, which is an essential early step during phagophore formation [9]. PI3K is regulated by the ULK1-complex consisting of the serine/threonine kinases ULK1, ULK2, FIP200, mAtg10 and Atg101 [7, 10].

Elongation of phagophores and the formation of autophagosomes depend on two ubiquitination-like reactions. First, Atg7 and Atg10 catalyze conjugation of the Ub-like protein Atg12 to Atg5 [11]. The conjugation product interacts with Atg16L1, and the ternary complex finally associates with the membrane of phagophores [12]. Second, ubiquitin-like molecules of the Atg8 family (LC3, GABARAP, and GATE-16) are modified by covalent addition of phosphoethanolamine (PE). In the case of LC3 addition of PE is catalyzed by Atg7, Atg3 and Atg12/Atg5-conjugate, which function as E1-like, E2-like and E3-like enzymes, respectively [13, 14]. Lipidated LC3 (LC3II) associates with the autophagosomal membrane where it promotes tethering and hemifusion during the formation of autophagosomes [7, 15]. Therefore, LC3II is commonly used as a marker of autophagy, but its deposition is not strictly confined to autophagosomal membranes.

## Selective autophagy of bacteria

Although starvation-induced autophagy appears to occur randomly in cells, autophagy of foreign bodies like invading bacteria (xenophagy) is obviously a selective process, which has become a subject of intense research. An early account of xenophagy, was the observation that *rickettsiae* accumulates in autophagosomal-like structures of guinea pig polymorphonuclear leukocytes [16]. The fate of microorganisms captured by autophagosomes is diverse: they may manipulate maturation of the autophagosome, escape into the cytosol, benefit from autophagy, or may be destroyed [17–22] .

Selective autophagy depends on adapter proteins, also termed SLRs (sequestosome-like receptors), because the best known adapter protein is p62/sequestosome1; other known members are NBR1, NDP52, and optineurin. SLRs comprise ubiquitin association regions (UBAs) and LC3 interacting regions (LIRs); they target bacteria to the autophagic pathway [23, 24]. Notably, p62 and NDP52 seem to serve non-redundant functions during selective autophagy [25, 26]. Modification of adapter proteins, for example, by phosphorylation adds specificity and another level of regulation to the process of selective autophagy [27, 28]. It remains unclear whether host proteins in the phagosomal membrane or bacterial proteins have to be ubiquitinated for targeting of SLRs. At any rate, ubiquitination seems not to be absolutely necessary for selective targeting of misfolded proteins to the autophagic degradation pathway. The BAG3-mediated chaperone-based targeting uses the specificity of Hsp70 chaperones to misfolded proteins as the basis for selectivity [29].

## Evidence for a role of membrane damage for selective autophagy

Involvement of membrane damage and/or pore-forming toxins in selective autophagy has emerged as a common theme from several studies of different bacterial species, namely *Streptococcus pyogenes, Listeria monocytogenes, Shigella flexneri*, *Salmonella* *enterica*, and *S. aureus* [21, 30–32]. Membrane damage/PFT might promote selective autophagy in several ways: first, by allowing bacterial escape from vacuolar compartments; second, *via* (modification and) recognition of pore-forming agents per se by the autophagy machinery; or third, *via* biochemical or biophysical changes secondary to pore formation which are recognized by the autophagy machinery, three possibilities that are mutually non-exclusive. Apparently, membrane remnants or damaged vacuoles are targeted by autophagy [33, 34], and growth of *Salmonella* in infected cells is reportedly restricted by autophagic targeting of damaged SCV (*Salmonella* containing vacuoles) [35, 36].

In the case of intracellular *Listeria* and intracellular *S. aureus*, it has been demonstrated that selective autophagy correlates with the ability of these bacteria to produce a pore-forming toxin (LLO and α-hemolysin, respectively), [31, 37, 38]. GFP-LC3 and p62 were also recruited to endocytosed toxin after application of purified protein or as a liposomal complex [37, 38]. This suggests that escape from vacuoles and subsequent exposure of bacteria to the cytosol is probably not required for selective autophagy to occur at intracellular *Listeria* or *S. aureus*; toxin appears to suffice as a trigger. Because LLO has been shown to be ubiquitinated, it is possible that a fraction of modified toxin binds adapter proteins and is internalized by autophagosomes instead of being delivered to proteasomes. In line with this, LLO has been shown to colocalize with p62 and ubiquitin in aggresome-like structures [39]. Similarly, *S. aureus* α-toxin colocalizes with p62 in target cells. Whether ubiquitination of toxin is required for the recruitment of p62 is currently unclear, but the available data suggest that pore-forming toxins can be ubiquitinated, which might suffice to directly recruit adapter proteins [40]. Alternatively, host proteins on damaged membranes could be ubiquitinated and recruit adapter proteins. The identification of ubiquitination targets, of the enzymes involved, and cues that actually initiate the process remains an important task. Antibodies specific for ubiquitin have been shown to label membrane remnants (defined by co-staining with antibodies directed against Galectin-3) in *Shigella*-infected cells. The authors proposed that membrane remnants are targeted by the autophagic machinery in a p62-dependent manner [33]. More recently, it was reported that the adapter protein NDP52 directly interacts with Galectin-8 which is recruited to the SCV (*Salmonella*-containing vesicles) of cells infected with *S. enterica* [34]. Thus, sugar moieties which become exposed after damage of vacuoles play a role for early recognition by adapter proteins. Because NDP52 and p62, despite structural similarity, seem to be functionally non-redundant and localize to distinct microdomains on vacuolar membranes/bacteria targeted for autophagy, it remains unclear how p62 is targeted to bacteria and/or membrane remnants.

Another pathway implicated in selective autophagy is based on the generation of the lipid second messenger diacylglycerol; it plays a role for xenophagy of *Salmonella* and does not depend on Atg5 [41].

It should be noted that although deployment of p62 is a hallmark of selective autophagy, p62 is also involved in starvation-induced autophagy [42, 43], providing a molecular link between random and selective autophagy.

## Perforation of the PM triggers a starvation response and autophagy

The earliest account of autophagy induction by a pore-forming toxin (PFT) was a report about the protective role of autophagy for target cells of *Vibrio cholerae* cytolysin, a small β-barrel PFT [44]. Subsequent work on the structurally related *S. aureus* α-toxin provided clues to the signaling pathways involved in autophagy induction by PFT and to the protective mechanism of autophagy: Previous studies with *S. aureus* α-toxin and SLO had established that membrane damage by these PFT, and consequences thereof are reversible in many cell types [45–49]. The transient nature of PM perforation is reflected by massive, but transient drop of cellular ATP levels, a fact that our group has exploited to characterize repair/recovery mechanisms. One important finding of these studies was that the removal of the oligomeric plasma membrane pore complexes by dynamin-dependent endocytosis is prerequisite for membrane repair after *S. aureus* α-toxin attack [50]. Second, membrane perforation turned out to trigger starvation and metabolic reprogramming, which proved to be also essential for recovery [38]. These findings were born out of an unbiased transcript profile of perforated cells obtained by serial analysis of gene expression (SAGE). This analysis revealed that membrane perforation by *S. aureus* α-toxin triggers expression of immediate early genes [48], whereby translation of these transcripts was delayed; moreover, eIF2α-phosphatase GADD34 was among the most abundant transcripts overexpressed under these conditions [48]. Together, this suggested that α-toxin causes an *integrated stress response, via* transient phosphorylation of eukaryotic translation initiation factor 2α, (eIF2α), and transient, global translational arrest. This assumption was confirmed, and GCN2 and PKR were subsequently identified as 2 kinases responsible for phosphorylation of eIF2α in response to α-toxin [38]. Importantly, pore-dead single amino acid mutants failed to activate this response, firmly establishing the link between membrane pore formation and *integrated stress response*. GCN2 is a sensor of amino acid starvation, which is conserved between yeast and man; uncharged tRNAs trigger autophosphorylation of GCN2. Activated GCN2 phosphorylates eukaryotic translation initiation factor 2α, leading to global translational attenuation [51, 52]. Intriguingly, GCN2 and PKR had previously been found to be required for amino acid starvation-induced autophagy [53]. Therefore, we reasoned that membrane perforation by PFT might also cause amino acid starvation, and that translational stop and autophagy might be required to overcome nutrient shortage during transient perforation.

Because exposure of susceptible cells to pore-forming toxins causes massive drop of intracellular ATP [45–49], we assumed that PFT activate AMPK. This kinase serves as the major cellular energy sensor, which regulates the activity of mTORC1, a master switch of translation and autophagy which integrates multiple signals from nutrient sensors. In fact, *S. aureus* α-toxin, *V. cholerae* cytolysin, Streptolysin O, and *E. coli* hemolysin induced phosphorylation of AMPK in epithelial cells [38]. Moreover, S6K, a substrate of mTORC1, became dephosphorylated. Transmission electron microscopy analyses revealed many multivesicular bodies, large, empty vacuoles, and vesicles delineated by double membranes, providing morphological evidence that PFT induced autophagy. Recently, it was found that mTORC1 activity is also regulated through availability of amino acids. Interestingly, amino acids induce relocalization of mTOR to lysosomal membranes in a p62-dependent way [42], suggesting that p62 may link selective and starvation-induced autophagy. That AMPK and GCN2 are activated by PFT suggested that damage of the plasma membrane causes cellular starvation and energy shortage, the classic inducers of autophagy. In fact, uptake of radio-labeled leucine was reduced by α-toxin [38], presumably because amino acid transporters were inhibited by the dissipation of natural ion gradients. Two recent studies appear to support our conclusion that membrane damage causes starvation and triggers nutrient sensors like GCN2/p-eIF2α and mTORC1: one paper reports that these pathways are also triggered by intracellular *Salmonella* and *Shigella*, and the authors propose that this is also due to membrane damage [54]; the second study indicates that the concept of pore-forming toxin-dependent activation of nutrient sensors holds in a Drosophila infection model [55].

## Loss of potassium links plasma membrane damage to activation of metabolic sensors

**Fig. 1:**
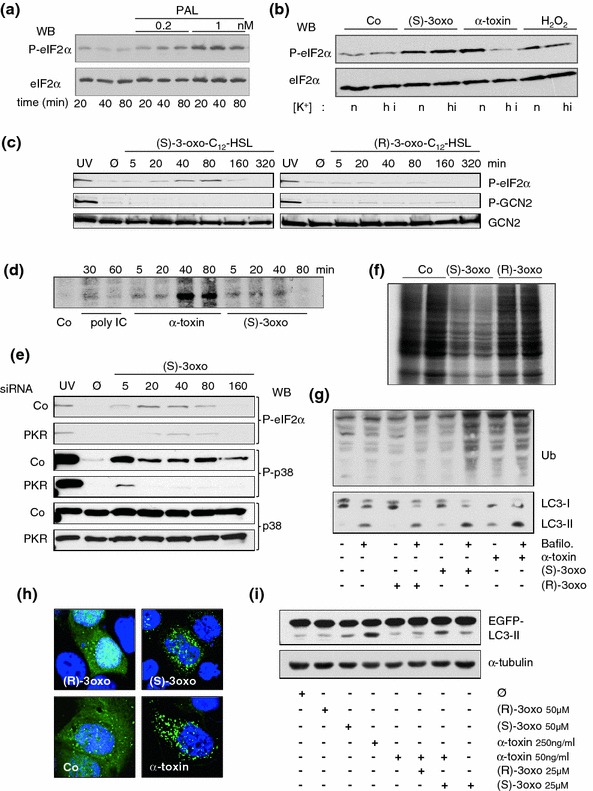
A quorum-sensing hormone of *P. aeruginosa* impacts translation and autophagy. Materials and methods employed here have been published previously [59, 88]. Metabolic labeling was performed as described by [89]. In the figure, (S)-3oxo and (R)-3oxo denote (S)-3-oxo-C12-HSL and (R)-3oxo-C12-HSL, respectively. **a** Western blot for (p)-eIF2α with whole cell lysates (HaCaT) treated with palytoxin (PAL) as indicated in the figure. **b** Western blot for (p)-eIF2α with whole cell lysates (HaCaT) treated with the indicated compounds in the presence of normal concentrations of potassium (n), or in media with high concentration of potassium (hi) [59]. **c** Western blot for p-eIF2α, p-GCN2, and GCN2 after treatment of HaCaT cells with the *P. aeruginosa* quorum-sensing hormone (S)-3-oxo-C12-HSL or the control compound (R)-3-oxo-C12-HSL for the indicated times. As expected, (R)-3-oxo-C12-HSL fails to cause phosphorylation of eIF2α. *Note* that GCN2 is not phosphorylated in response to either lactone; UV served as a positive control. Untreated cell samples (*media alone*) are denoted Ø. **d** Autoradiographic detection of P^32^-PKR in samples of HaCat cells treated for the indicated times with the compounds denoted underneath the panel. Co: medium alone. **e** Western blots for p-eIF2α, p-p38, or p38 with straight Cos7-cell lysates obtained 48 h following transfection with siRNAs and subsequent treatment with S-3-oxo-C12-HSL. **f** Autoradiography of an SDS-Gel visualizing incorporation of S^35^-Methionine into newly synthesized proteins. *Note* marked inhibition of protein synthesis in cells treated with S-3-oxo-C12-HSL. **g** Western blots for ubiquitination and LC3I/II with lysates of HaCat cells treated as indicated in the figure for 3 h. **h** Fluorescence microscopy images of HaCat cells transfected with EGFP-LC3 and treated for 3 h with compounds indicated in the figure. *Note* redistribution of diffuse green fluorescence signal into dots in cells treated with α-toxin, or S-3-oxo-C12-HSL. **i** Western blot for GFP with lysates of HaCat cells transfected with EGFP-LC3 treated as indicated in the figure. The untreated cell sample (*media alone*) is denoted Ø. Loading control with α-tubulin. Combination of (S)-3-oxo-C12-HSL and α-toxin led to a significant accumulation of LC3II

Rapid release of potassium ions is a common consequence of membrane perforation by PFT, for example [56, 57]. Loss of potassium—or low concentrations of this ion in perforated cells—seems to trigger a multitude of responses in target cells of PFT, because high concentrations of potassium in the extracellular milieu prevent these responses, and because they can be also triggered by the potassium ionophore nigericine. Examples are the activation of caspases [56, 58], activation of kinases including, for example, p38 [59], AMPK, GCN2 [38], and CREB [60]. Importantly, high potassium concentrations in media did not impair activation of p38 by hydrogen peroxide, indicating that these experimental conditions do not generally paralyze pathways leading to the phosphorylation of stress kinases [38]. That GCN2 is activated by membrane perforation in a potassium-efflux-dependent manner raises the possibility that cells exploit the dependence of nutrient transport across the PM on physiological ion gradients to indirectly sense changes of ion concentrations. The link between physiologic ion gradients and regulation of translation initiation has been noted previously in a paper about the marine toxin Palytoxin (PAL) which converts the Na^+^/K^+^ exchanger to a channel. As a consequence, intracellular potassium concentrations drop and translation stops [61]. In line with this, PAL causes phosphorylation of eIF2α (Fig. 1a).

## The role of MAPK for cellular responses to pore-forming toxins: potential links to autophagy

Several pathways have been implicated in the survival of cells or whole organism (*Caenorhabditis elegans* and insects) exposed to PFT or microorganisms releasing PFT [44, 57, 62–69]; for review, see [70–73]. Among them, the p38 MAPK pathway was the first found to protect *C. elegans* or mammalian cells from small pore-forming toxin attack [66]. Subsequently, we demonstrated in mammalian cells that p38 does not confer resistance to the pore-forming activity of *S. aureus* α-toxin, but is required for recovery from perforation. It was also demonstrated that this function was not required for recovery from membrane perforation by a large pore-forming toxin (SLO), revealing that membrane repair or metabolic recovery mechanisms are diverse [67]. At low concentrations, α-toxin does not induce a significant influx of Ca^++^ ions, and therefore would not be able to trigger the so-called wounded membrane response [74, 75]. Although the critical downstream targets of p38 for recovery from α-toxin are not known to date, the finding that α-toxin has to be internalized by target cells and that autophagy is required for energy homeostasis offers at least two potential explanations: Because Rab5 is a regulator of endocytosis [76], and because p38 impacts on the engagement of Rab5 with membranes [77], it is possible that p38 is required for the internalization of small PFT. MAPK p38 has also been implicated in regulation of autophagy [78, 79]. Therefore, it is possible that one way this kinase may impact on the outcome of an attack by PFT is through regulation of autophagy. JNK, another important stress-activated protein kinase (SAPK) is also activated by PFT and may therefore impact cellular responses to membrane perforation [68]. An important role of JNK for starvation-induced autophagy has been demonstrated [80].

## A bacterial quorum-sensing hormone of *P. aeruginosa* attenuates translation and impacts autophagy

Apart from PFT, few other bacterial exoproducts including LPS [81] and NLR ligands [82–84] have been shown to induce autophagy. (S)-3-oxo-C12-HSL is a small molecule produced by the Gram-negative bacterium *Pseudomonas aeruginosa*. (S)-3-oxo-C12-HSL is not only involved in regulating cell-to-cell signaling in these bacteria, but impacts signaling in mammalian cells [85]. Thus, (S)-3-oxo-C12-HSL has been shown to inhibit NF-κB [86] and oxidative stress [87], and to trigger phosphorylation of p38 MAPK and eIF2α [88]. Because the latter two events are also triggered by PFT, and because eIF2α appears to be important for the induction of autophagy in response to *S. aureus* α-toxin, we have recently started to investigate whether (S)-3-oxo-C12-HSL might also impact on autophagy. First, we confirmed that (S)-3-oxo-C12-HSL triggered eIF2α-phosphorylation in HaCat cells. Notably, high concentration of potassium in media did not affect phosphorylation in response to (S)-3-oxo-C12-HSL, although it blocked phosphorylation of eIF2α in perforated cells (Fig. 1b). (S)-3-oxo-C12-HSL did not cause phosphorylation of eIF2α-kinase GCN2 (Fig. 1c), but led to the phosphorylation of eIF2α-kinase PKR (Fig. 1d). Therefore, signals upstream of phosphorylation of eIF2α after treatment with PFT, or (S)-3-oxo-C12-HSL are different. Although (S)-3-oxo-C12-HSL-dependent phosphorylation of PKR was weaker than that caused by α-toxin (Fig. 1d), knock down of PKR inhibited (S)-3-oxo-C12-HSL-dependent phosphorylation of eIF2α, indicating that PKR plays a critical role for cellular stress signaling in response to this compound (Fig. 1e). In line with this, activation of p38 was also markedly reduced after KD of PKR (Fig. 1e). Unlike PFT, (S)-3-oxo-C12-HSL did not cause significant loss of ATP at early time points, and only moderate dephosphorylation of S6K was discerned (data not shown). Thus, metabolic changes in response to (S)-3-oxo-C12-HSL appear to be less pronounced than those caused by PFT. However, metabolic labeling experiments revealed that (S)-3-oxo-C12-HSL inhibits translation (Fig. 1f). Many stress responses are accompanied by enhanced ubiquitination and degradation of proteins. Therefore, we investigated ubiquitination of proteins in (S)-3-oxo-C12-HSL-treated cells (Fig. 1g). In contrast to α-toxin, (S)-3-oxo-C12-HSL alone appeared not to significantly enhance steady state levels of ubiquitinated proteins, but addition of bafilomycin revealed that (S)-3-oxo-C12-HSL increased the rate of ubiquitination. In Western blot experiments with antibodies against LC3, higher levels of LC3II were observed following treatment with α-toxin or (S)-3-oxo-C12-HSL, and in both cases, this effect was enhanced by bafilomycin (Fig. 1g, lower panel). Consistently, numbers of GFP-LC3-positive puncta in transiently transfected cells were increased by (S)-3-oxo-C12-HSL (Fig. 1h). Therefore (S)-3-oxo-C12-HSL impacts autophagy. Since *P. aeruginosa* and *S. aureus* are commonly found together in wounds, or in the respiratory tract of patients suffering from cystic fibrosis, potential synergism of (S)-3-oxo-C12-HSL and *S. aureus* α-toxin might bear on the pathogenesis in coinfections with these bacteria. In fact, the combination of (S)-3-oxo-C12-HSL and *S. aureus* α-toxin at concentrations which caused little increases in LC3II, led to a significant accumulation of this product (Fig. 1i).

## Conclusion

**Fig. 2:**
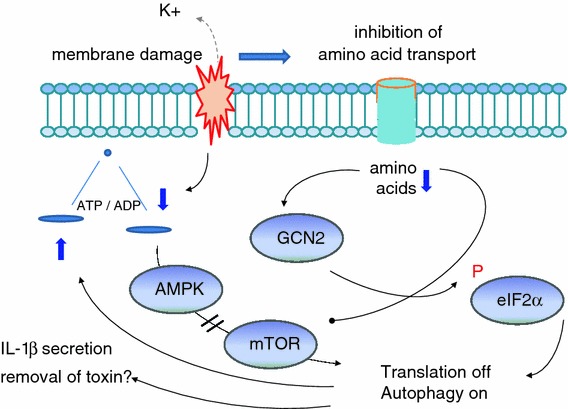
Membrane damage by pore-forming toxins induces classic starvation responses. PFT represents an important class of bacterial exoproducts which affect host-cell translation and autophagy. Loss of cellular potassium from perforated cells leads to the failure of nutrient transport and transient drop of ATP, thus activating cellular nutrient and energy sensors GCN2 and AMPK, subsequent phosphorylation of eIF2α and deactivation of mTORC1. As a consequence, transient global translational attenuation and induction of starvation-associated autophagy occur to overcome energy and nutrient crisis caused by membrane damage. Membrane perforation by α-toxin induces IL-1β secretion [56], and a role of autophagy in unconventional secretion of IL-1β has been recently documented [5]. Whether autophagy is also involved in the release of toxosomes [50], undigestible toxin oligomers associated with exosomal-like structures, remains to be investigated

Bacterial exoproducts may impact host-cell translation and autophagy. Pore-forming toxins (PFT) represent one important class of bacterial exoproducts which affect these processes. Based on the studies of cellular responses to PFT, including *S. aureus* α-toxin, we found that the loss of cellular potassium from perforated cells leads to failure of nutrient transport and loss of ATP, thus activating cellular nutrient and energy sensors GCN2 and AMPK, subsequent phosphorylation of eIF2α and deactivation of mTORC1 (Fig. 2). Therefore, we propose that nutrient/energy sensors serve as sentinels of membrane integrity. In addition to the removal of membrane pores by endocytosis, phosphorylation of eIF2α and induction of autophagy by these pathways are required to prevent abysmal loss of ATP in cells perforated by *S. aureus* α-toxin. Thus, studying transient membrane perforation by *S. aureus* α-toxin has provided important novel insights into cell autonomous defense against an archaic threat to a cell, namely damage of the plasma membrane.

Like PFT, (S)-3-oxo-C12-HSL, a quorum-sensing hormone of *P. aeruginosa* causes phosphorylation of eIF2α, attenuation of translation, and accumulation of autophagosomes. However, (S)-3-oxo-C12-HSL did neither cause severe loss of ATP nor phosphorylation of GCN2; and phosphorylation of p-eIF2α was insensitive to high levels of extracellular potassium. Therefore, PFT and (S)-3-oxo-C12-HSL modulate translation and impact autophagy by different pathways, which may act synergistically.
